# Virtually Administered Intervention Through Telerehabilitation for Chronic Non-specific Low Back Pain: A Review of Literature

**DOI:** 10.7759/cureus.42942

**Published:** 2023-08-04

**Authors:** Priti Mehendale, Madhavan Iyenagar, Geeta Bhatt, Shweta Manwadkar

**Affiliations:** 1 Department of Physiotherapy, Parul Institute of Doctoral Studies, Parul University, Vadodara, IND; 2 Department of Surgery, Parul Institute of Doctoral Studies, Parul University, Vadodara, IND; 3 Department of Neurophysiotherapy, K.J. Somaiya College of Physiotherapy, Mumbai, IND; 4 Department of Cardiorespiratory Physiotherapy, K.J. Somaiya College of Physiotherapy, Mumbai, IND

**Keywords:** electronic health, kinesiophobia, exercise therapy, chronic non-specific low back pain, telerehabilitation

## Abstract

The most frequent reason for individuals to seek medical attention in both primary care settings and immediate care centers is low back pain (LBP). Over a duration of time, the disability caused by lower back pain has risen enough, particularly in countries with low or moderate incomes. In the coming years, there may be an increase in LBP-related impairment and expenses in countries with low or medium incomes, particularly when fragile medical systems are unable to handle this growing load. Hence, this review focuses on the effectiveness of telerehabilitation (TR) on LBP. The significant advantages of TR may include greater interaction and remote accessibility to medical treatments. The exchange of knowledge and health information is made possible through a more effective interaction, which benefits patients, families, carers, physicians, and researchers. People who live in distant places now have the opportunity to get medical attention assisting families in caring for patients with poor responsiveness. In addition, it provides the potential for prompt detection, the beginning of treatment in the midst of an emergency, a shorter stay in the hospital, ongoing monitoring of those at risk, and overall time and expense savings. Therefore, this study supports the application of TR in conditions of LBP for early management and relief of pain for patients in low-resource areas.

## Introduction and background

Low back pain (LBP) is defined as pain or discomfort felt below the costal margin and above the inferior gluteal folds, with or without leg pain. One of the most frequent reasons for patients visiting both in a primary care environment and in an emergency room is back pain. According to individuals' demographics, the aetiologies might vary, although mechanical or non-specific causes account for the majority of cases [1]. Between 1990 and 2017, the incidence and year lived with disability (YLD) rates of LBP declined marginally at the global level. Females had a higher incidence of YLDs than males. YLD occurrence rises with age and peaks between the ages of 35 and 49. LBP continues to be the main contributory factor of YLDs globally, but it is still not sufficiently recognized as an illness load in the community, with a significant gap still existing between the amount of incidence and the response of policy, research, and medical facilities [2].

Telerehabilitation (TR) use has increased significantly in developed nations, although the field is still relatively new. In comparison to conventional hospital or person-to-person treatments, TR generally lowers expenses for medical professionals and patients. In addition, this sort of innovation might help patients who reside in rural areas where typical therapy programs might not be readily available. Nonetheless, it is important to not undervalue some of the drawbacks of TR, such as patients' skepticism about communicating with their doctors or therapists through the Internet [2].

As per previous studies, LBP is increasing rapidly due to sedentary lifestyles, lack of awareness regarding good posture, and negligence toward its management due to expensive rehabilitation. Therefore, this review focuses mainly on the importance of TR in LBP conditions along with its beneficial effects.

## Review

Search methodology

**Figure 1:**
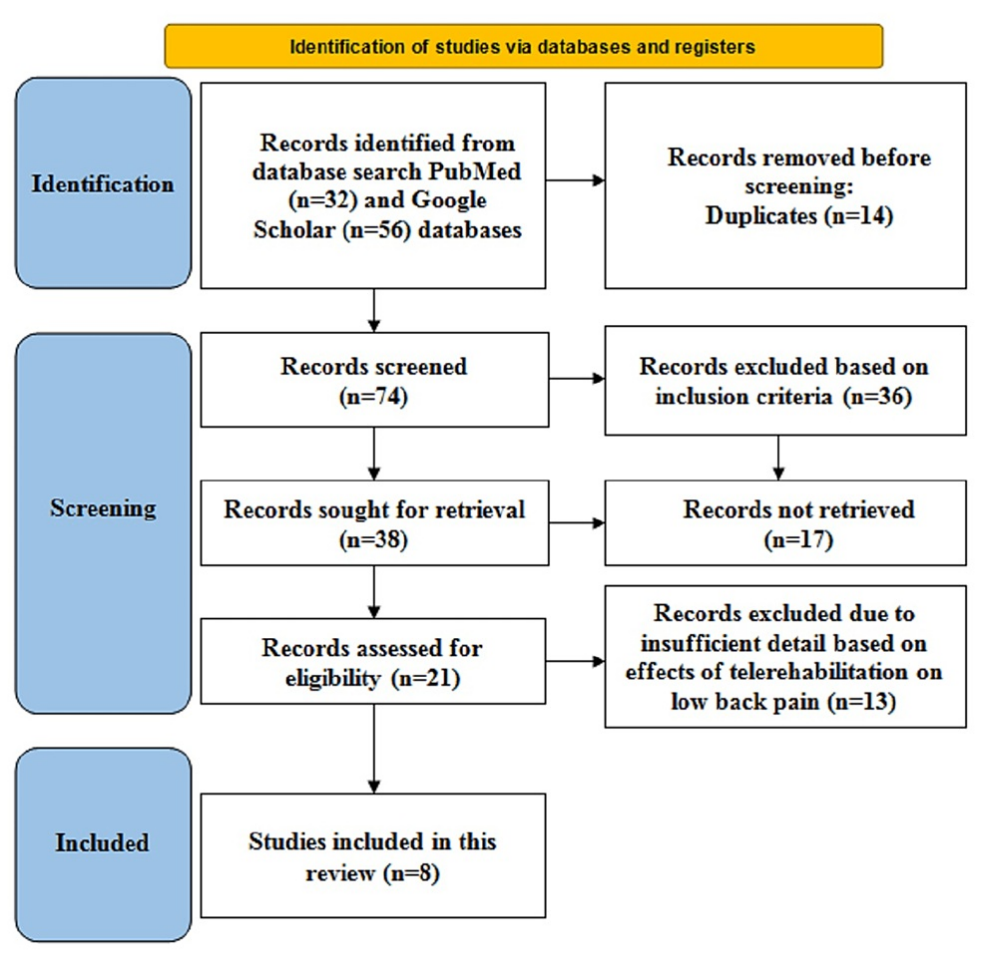
Search strategy in accordance with PRISMA guidelines PRISMA: Preferred Reporting Items for Systematic Review and Meta-Analysis

The search methodology involved searching PubMed and Google Scholar databases for articles that were published between 2018 and 2023. The keywords that were used to search the articles included “telerehabilitation” and “low back ache.” The inclusion criteria consisted of articles with full-text availability, articles giving information regarding the effects of TR on LBP, studies that included only humans, and articles in the English language. The articles not providing brief descriptions regarding the effects of TR on LBP, studies performed on animals, and articles that were not published in the English language were excluded from the study. The articles were screened based on the inclusion and exclusion criteria, and a total of eight articles were included in this review. This review followed Preferred Reporting Items for Systematic Review and Meta-Analysis (PRISMA) guidelines for search strategy, as illustrated in Figure 1.

Data extraction

Data consisting of the author/s and year, study design, methodology, and summary of the findings incorporated in this review is briefly described in Table 1. 

**Table 1:** Summary of the studies included in this review TR: telerehabilitation; LBP: low back pain; DCP: digital care program; RCT: randomized controlled trial

Serial no.	Author and year	Aim of the study	Methodology	Conclusion
1	Fatoye et al. (2022) [3]	To assess the difference between TR and a clinic-based treatment for Nigerians with non-specific chronic LBP in terms of patient benefit and financial efficacy	There was a randomized controlled study. Either TR-based McKenzie therapy or clinic-based McKenzie therapy was given to non-specific chronic LBP sufferers. For eight weeks, treatment was conducted three times each week. The Oswestry Impairment Index (ODI) was utilized to assess patients' levels of impairment at baseline, week four, and week eight. The ODI was attached to the short-form six-dimension tool to produce quality-adjusted life years, which were used to calculate the patients' health-related quality of life estimates. In Nigeria, in 2019, healthcare utilization of assets and expenditures were evaluated using the McKenzie extension procedure.	TR was less expensive for those with non-specific LBP.
2	Seron et al. (2021) [4]	To enumerate the data that are accessible on TR in physiotherapy (PT) through systematic reviews	The review involved searching of Medline/PubMed, EMBASE, and Cochrane Library databases. The treatment has to be TR by PT, which can be described as the delivery of rehab with measures in any field of PT done virtually instead of regular visits by an instructor far from the client's location and utilizing telecommunications technology.	For illnesses, including osteoarthritis, LBP, hip and knee replacement, and multiple sclerosis, along with cardiac and pulmonary rehabilitation, TR in PT may be similar to conventional treatments or more effective than no treatment.
3	Bailey et al. (2020) [5]	To assess the effectiveness of a 12-week digital care program (DCP) in a sizable group of individuals with persistent back and knee pain	It was a longitudinal observational study through a mobile app-based remote DCP with 10,264 patients. The subjects took part in a 12-week multimodal DCP that included education, exercise therapy (ET), and one-on-one remote health teaching to enhance behavioral wellness. The primary outcome measure included was a visual analogue scale (VAS) to measure pain intensity.	These outcomes indicate the effectiveness and scalability of a DCP for treating persistent LBP and knee pain in a greater, diverse, and actual community. There was a strong positive connection between participation and alleviating pain, and subjects showed high completion and participation rates.
4	Werneke et al. (2022) [6]	To evaluate functional status (FS) results by TR delivery modality for people with LBP and to investigate relationships between the frequency of TR and the results of FS, number of visits, and feedback from patients throughout COVID-19	A comprehensive national patient dataset with information regularly gathered in outpatient physical rehabilitation clinics was examined in an observational retrospective analysis. Patients provided information on the average number of TR visits during every session using survey-based questions that were given out throughout the period of service.	The findings showed that for those with LBP treated by physiotherapists in outpatient settings during the COVID-19 epidemic, using TR was linked with identical results to standard in-person therapy.
5	de la Cal et al. (2021) [7]	To investigate the benefits and drawbacks of establishing a web-based TR approach for managing chronic LBP among physiotherapists	The qualitative research included 19 physiotherapists from clinical and educational institutions in the public and commercial sectors. To ascertain the subjects' prevalent viewpoints, articles were taken from a transcript of semi-structured, one-on-one, thorough conversations with each consenting subject. Every question-and-answer session remained for around 40 minutes.	The findings imply that clients must actively participate in their own care in order for TR to be effective. Exercise regimens for LBP, however, are not typically tailored to individual desires. The follow-up and remote interaction that individuals seek from physical therapists are now possible owing to new technologies, but patients' long-term commitment to therapy depends on their understanding of the exercises and the right methods to use when performing them.
6	Hou et al. (2019) [8]	To evaluate the effectiveness of mobile phone-based therapy programs in lumbar spinal surgery patients	Individuals who had spinal operations were included in an RCT and randomly assigned to receive either the typical course of care that consists of conventional in-person therapy or an eHealth (electronic health) program that was accessible via a mobile phone.	This study established the efficacy of a telephone-based TR program for after-surgery LBP patients' self-managed therapy. Individuals who had better compliance levels showed increased benefits of eHealth.
7	Mbada et al. (2019) [9]	The outcomes of TR-based McKenzie therapy (TBMT) and clinic-based McKenzie therapy (CBMT) for LBP patients were compared in this research.	This quasi-experimental research was conducted by 47 willing individuals with persistent LBP who showed a "directional bias" for the McKenzie extension protocol (MEP). Using block-permuted randomization, the individuals were divided into the CBMT or TBMT groups. For eight weeks, three times per week, individuals in the CBMT and TBMT groups had MEP, which involved a precise series of lumbosacral repetitive motions in extension intended to centralize, reduce, or eradicate clinical features. The results were evaluated in terms of pain intensity (PI), back extensors' muscle endurance (BEME), activity limitation (AL), participation restriction (PR), and general health status (GHS) during the fourth and eighth weeks of the trial.	The results of the McKenzie extension protocol used in TR are equivalent to those of the clinic-based McKenzie treatment. Thus, the McKenzie extension used in TR is useful in managing individuals with non-specific persistent LBP. Therefore, McKenzie therapy delivered via TR may be able to fill the gap left by the absence of clinic-based McKenzie therapy facilities, particularly in distant areas.
8	Dias et al. (2021) [10]	To find out if TR based on exercise helps persons with physical impairments with their pain, motor skills, and standard of living	Studies that assessed exercise using TR were taken into consideration. Control and other treatments were used as comparisons. Pain, physical function, and quality of life were the results. Study selection, data extraction, and analysis adhered to the PROSPERO methodology.	When compared to other interventions, exercise through TR may be a choice to manage pain, function in the body, and quality of life in persons with physical impairments.

Discussion

This review highlights the effects of TR in patients with LBP compared to in-person rehabilitation and other therapies. The evidence regarding the effectiveness of exercise therapy in the management of LBP is diverse. Therefore, in this review, exercise therapy through TR was considered for the management of LBP that reported better pain and functional outcomes [11-13]. Kothari et al. concluded that individuals with mechanical LBP can benefit from traditional physical therapy in terms of lowering pain, kinesiophobia, and impairment [11]. Based on a previous study, rehabilitation through in-person exercise therapy stays the primary therapy for persistent LBP and should be applied often [14]. According to Dagenais et al., physiotherapy and in-patient healthcare accounted for the biggest share of overall healthcare expenditures for LBP [15]. For various musculoskeletal issues, and in relation to cardiac and pulmonary therapy, TR in physiotherapy may be very similar to conventional treatment or more beneficial in comparison to no management at all [4,16]. The better therapies for individuals with non-specific chronic LBP are pilates, stabilization/motor control, resistance training, and aerobic exercise training. In addition, exercise instructions along with manual therapy and supervision from the therapist increase the positive impact of the treatment [17].

A study reported the efficacy of a phone-based TR method for treating LBP that occurred after surgery and is self-managed therapy and concluded that individuals who had better compliance levels showed increased benefits of eHealth [8]. Other studies reported that using TR in outpatient clinics for people with LBP under the supervision of therapists during the COVID-19 epidemic was related to identical results in comparison to standard in-person therapy [6,18]. Few studies have found that, in terms of the functioning level and quality of life, TR programs are equally practical and effective as conventional physiotherapy [10]. In addition, TR provided better relief and satisfaction in terms of follow-up outcomes [19,20].

According to the findings of a previous study, TR can only be effective if individuals take initiative in their personal care. Exercise regimens for LBP are not based on the patient's preferences in TR but enable therapists to offer clients the ongoing supervision and distance communication required along with long-term adherence to therapy depending on the client's comprehension of the exercises and the proper procedures used by those exercises [7]. Some contrasting findings demonstrated that present-day digital health therapies independently do not reduce pain and impairment in chronic LBP in comparison to conventional basic interventions, whereas some trials have shown early evidence that telemedicine may be an addition to standard therapy for patients with acute and subacute LBP. Moreover, assessing the efficacy of telehealth-based therapies depends on larger, higher-quality research with prolonged follow-ups [21].

According to Özden et al., the TR group saw noticeable improvements in pain, activity, quality of life, kinesiophobia, satisfaction, and motivation after eight weeks of therapy [22]. Furthermore, in relation to the traditional therapy group, the TR group showed more beneficial effects in all the outcome measures. Clinical metrics and rehabilitation adherence among individuals with LBP are positively impacted by the video exercise-based TR program [22].

A multi-step procedure was used to make the switch to telehealth physical therapy, involving educating clinicians, creating protocols and instructions, and learning more about the patient experience. Physiotherapy delivered via telehealth has already demonstrated advantages, including greater accessibility to treatment and improved patient consistency. High-value telehealth rehabilitation still has a position in treating individuals who have musculoskeletal disorders even after the presence of a public health crisis [23]. According to Werneke et al., TR is an effective kind of therapy for people with LBP and provided beneficial outcomes during the COVID-19 period [6].

In the present review, it was observed that TR shows almost similar or equal results in comparison to clinical-based rehabilitation and is a more convenient and cost-effective mode of rehabilitation in patients with LBP. From the COVID-19 pandemic, the TR mode improved to a certain extent to reduce the lacuna and betterment of services in physiotherapy practices, and this advanced method of treatment is beneficial and accessible to every individual irrespective of their location [6].

While TR can be a beneficial method of rehabilitating patients with chronic LBP, there are certain downsides. Elderly individuals may have a harder time navigating TR, and several studies have addressed how acceptance, usefulness, tolerance, and impacts of visual reality vary by age. A lengthy session time (recommended not to go over 30 minutes) and probable lag in the software's execution of physical exercise programs are two factors that might affect dizziness and nausea, which are both considered symptoms of "virtual reality sickness" [6,22].

The therapy of TR is still developing but offers the opportunity to create several intriguing paradigms for recovery that balance both expenses and benefits. The potential to lengthen and intensify treatment programs is one of the primary opportunities. It may be possible to lengthen the duration of the rehabilitation regimen and take advantage of modern technology. For example, one can increase training by monitoring patients' rehabilitation via telephone conferences or by using robotic or electronic equipment. To make this novel feature a standard therapy, there is a need for treatment facilities to change on the basis of this intervention as this advanced method of treatment is beneficial and accessible to every individual irrespective of their location.

## Conclusions

The review of the studies highlights that TR administered through a mobile application, web-based or phone-based, is extremely beneficial in the treatment of LBP, regardless of a few research contradicting the same. In addition to LBP, other illnesses can be cured with the help of TR, such as osteoarthritis and hip and knee replacement surgeries. Furthermore, during the COVID-19 pandemic, TR proved to be a boon for the rehabilitation process and is also less expensive in comparison to in-person rehabilitation. The functional outcomes resulted in fewer visits of patients when care was provided through TR and has a more positive influence on patients’ painful symptoms, quality of life, motivation, satisfaction, expectation, and kinesiophobia.

## References

[1] Koes BW, van Tulder MW, Thomas S (2006). Diagnosis and treatment of low back pain. BMJ.

[2] Wu A, March L, Zheng X (2020). Global low back pain prevalence and years lived with disability from 1990 to 2017: estimates from the Global Burden of Disease Study 2017. Ann Transl Med.

[3] Fatoye F, Gebrye T, Fatoye C, Mbada CE, Olaoye MI, Odole AC, Dada O (2020). The clinical and cost-effectiveness of telerehabilitation for people with nonspecific chronic low back pain: randomized controlled trial. JMIR Mhealth Uhealth.

[4] Seron P, Oliveros MJ, Gutierrez-Arias R (2021). Effectiveness of telerehabilitation in physical therapy: a rapid overview. Phys Ther.

[5] Bailey JF, Agarwal V, Zheng P, Smuck M, Fredericson M, Kennedy DJ, Krauss J (2020). Digital care for chronic musculoskeletal pain: 10,000 participant longitudinal cohort study. J Med Internet Res.

[6] Werneke MW, Deutscher D, Hayes D, Grigsby D, Mioduski JE, Resnik LJ (2022). Is telerehabilitation a viable option for people with low back pain? Associations between telerehabilitation and outcomes during the COVID-19 pandemic. Phys Ther.

[7] Martínez de la Cal J, Fernández-Sánchez M, Matarán-Peñarrocha GA, Hurley DA, Castro-Sánchez AM, Lara-Palomo IC (2021). Physical therapists' opinion of e-health treatment of chronic low back pain. Int J Environ Res Public Health.

[8] Hou J, Yang R, Yang Y (2019). The effectiveness and safety of utilizing mobile phone-based programs for rehabilitation after lumbar spinal surgery: multicenter, prospective randomized controlled trial. JMIR Mhealth Uhealth.

[9] Mbada CE, Olaoye MI, Dada OO (2019). Comparative efficacy of clinic-based and telerehabilitation application of mckenzie therapy in chronic low-back pain. Int J Telerehabil.

[10] Dias JF, Oliveira VC, Borges PR (2021). Effectiveness of exercises by telerehabilitation on pain, physical function and quality of life in people with physical disabilities: a systematic review of randomised controlled trials with GRADE recommendations. Br J Sports Med.

[11] Kothari P, Palekar T, Shah M, Mujawar S (2019). Effects of conventional physiotherapy treatment on kinesiophobia, pain, and disability in patients with mechanical low back pain. J Dent Res.

[12] Cottrell MA, Hill AJ, O'Leary SP, Raymer ME, Russell TG (2018). Clinicians' perspectives of a novel home-based multidisciplinary telehealth service for patients with chronic spinal pain. Int J Telerehabil.

[13] Hayden JA, Ellis J, Ogilvie R, Malmivaara A, van Tulder MW (2021). Exercise therapy for chronic low back pain. Cochrane Database Syst Rev.

[14] Shipton EA (2018). Physical therapy approaches in the treatment of low back pain. Pain Ther.

[15] Dagenais S, Caro J, Haldeman S (2008). A systematic review of low back pain cost of illness studies in the United States and internationally. Spine J.

[16] Suso-Martí L, La Touche R, Herranz-Gómez A, Angulo-Díaz-Parreño S, Paris-Alemany A, Cuenca-Martínez F (2021). Effectiveness of telerehabilitation in physical therapist practice: an umbrella and mapping review with meta-meta-analysis. Phys Ther.

[17] Owen PJ, Miller CT, Mundell NL (2020). Which specific modes of exercise training are most effective for treating low back pain? Network meta-analysis. Br J Sports Med.

[18] Dadarkhah A, Rezaimoghadam F, Najafi S, Mohebi B, Azarakhsh A, Rezasoltani Z (2021). Remote versus in-person exercise instruction for chronic nonspecific low back pain lasting 12 weeks or longer: a randomized clinical trial. J Natl Med Assoc.

[19] Muñoz-Tomás MT, Burillo-Lafuente M, Vicente-Parra A, Sanz-Rubio MC, Suarez-Serrano C, Marcén-Román Y, Franco-Sierra MÁ (2023). Telerehabilitation as a therapeutic exercise tool versus face-to-face physiotherapy: a systematic review. Int J Environ Res Public Health.

[20] Amin J, Ahmad B, Amin S, Siddiqui AA, Alam MK (2022). Rehabilitation professional and patient satisfaction with telerehabilitation of musculoskeletal disorders: a systematic review. Biomed Res Int.

[21] Dario AB, Moreti Cabral A, Almeida L (2017). Effectiveness of telehealth-based interventions in the management of non-specific low back pain: a systematic review with meta-analysis. Spine J.

[22] Özden F, Sarı Z, Karaman ÖN, Aydoğmuş H (2022). The effect of video exercise-based telerehabilitation on clinical outcomes, expectation, satisfaction, and motivation in patients with chronic low back pain. Ir J Med Sci.

[23] Grundstein MJ, Fisher C, Titmuss M, Cioppa-Mosca J (2021). The role of virtual physical therapy in a post-pandemic world: pearls, pitfalls, challenges, and adaptations. Phys Ther.

